# Healthy Navajo Women: Walk in Beauty

**Published:** 2006-06-15

**Authors:** Ramah Band of Navajo Indians and the Albuquerque Area Indian Health Board

## About the Video

The Ramah Band of Navajo Indians and the Albuquerque Area Indian Health Board produced a health promotion video to build community awareness of breast and cervical cancer.  Objectives of the video were to raise awareness of the burgeoning incidence of breast and cervical cancer among American Indian people, increase knowledge of breast and cervical cancer early detection exams, encourage community members to speak with their providers about cancer, and celebrate the strength of Ramah Navajo women and their importance in protecting community heritage.  Key cultural considerations (i.e. story telling, symbolic landmarks, and indigenous language) were incorporated into the production to bolster authenticity.

**Figure:**
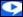
Watch* Walk in Beauty* (RM 9Mb) You will need RealOne Player to view these videos. Learn more about RealPlayer. Learn more about RealPlayer.

## Transcript

**Opening screen reads:**

Healthy Navajo Women Walk in Beauty

Produced by Ramah Band of Navajo Indians and

Albuquerque Area Indian Health Board, Inc.

Spoken in Navajo by a narrator, but caption on screen in English reads:

Through this video we will be discussing women's health, which all women should be aware of. Some of you are aware of cancer and some of you are not aware. Women have many responsibilities such as keeping a home, taking care of children and tending to sheep. We all need to be aware of cancer and to learn more about it. You will be able to understand what cancer is and how to detect it throughout this video.

**Jolene Luna, MS, Health Educator**

Indian women today face many challenges and responsibilities, which prevent us from taking care of our bodies. Unfortunately, cancer has become a major health concern for all Indian people. In fact, cancer has become the second leading cause of death for Indian women. Two types of cancer that affect women are becoming very common in our communities. They are breast and cervical cancer. However, this is not a hopeless situation. There is something that each of us can do. We can go to the clinic for our annual Pap exams and mammograms. When we do this, we can find breast and cervical cancer early, which prevents it from spreading throughout our bodies and improves the chances of treating it and beating it. As women, we are the lifeblood of the community. We need to make sure that we have our exams each year to keep our families together to protect our heritage.

**Final screen reads:**

Keep our Heritage Alive

Make an appointment for your well woman's exam today!

## Author Information

For more information about the video, please contact Carolyn Finster, Pine Hill Health Center, 505-775-3271

